# Esophageal Perforation After Failed Prehospital Intubation

**DOI:** 10.5811/cpcem.2018.6.38088

**Published:** 2018-07-16

**Authors:** Kaila Pomeranz, Nicholas Mohr

**Affiliations:** University of Iowa Hospitals and Clinics, Department of Emergency Medicine, Iowa City, Iowa

## Abstract

Esophageal perforation is a rare condition with high rates of mortality if not recognized quickly. This is a case of a 67-year-old male with a self-inflicted gunshot wound to the head. He had one failed intubation attempt prior to arrival. On postmortem autopsy it was discovered that in addition to significant head trauma he also had an esophageal and gastric rupture.

## INTRODUCTION

Esophageal perforation occurs rarely, with an estimated 3.1/1,000,000 population per year.^1^ Inciting events are typically iatrogenic or spontaneous rupture and, less commonly, trauma. Even with prompt diagnosis and recognition, mortality rates approach 20%. Gastric contents leak out from the site of rupture, leading to significant inflammation and infection.^1^ Esophageal perforation can occur due to unrecognized esophageal intubation or improper bagging techniques.^2^ While uncommon, these injuries are likely under-reported even when recognized, and can have serious implications if not identified. Few case reports cite this complication, and none previously attributed esophageal rupture to recognized prehospital esophageal intubation.

## CASE REPORT

A 67-year-old male with past medical history only of hypertension was brought to the emergency department (ED) after a suspected self-inflicted gunshot wound to the head approximately 30 minutes prior to arrival. The patient was found supine and unresponsive by emergency medical services (EMS) with stridorous breath sounds. Paramedics attempted intubation once, but after recognizing esophageal intubation through auscultation they removed the endotracheal tube and placed a King laryngeal tube (LT) supraglottic airway (Ambu®). The second attempt was confirmed by auscultation of bilateral breath sounds and digital end-tidal carbon dioxide monitoring. The airway was suctioned through the King LT and 200 mL of blood was removed. Initial vital signs at the scene were pulse 77 beats per minute (bpm), blood pressure (BP) 134/63 millimeters of mercury (mmHg), room air oxygen saturation (SaO_2_) 70%.

Upon arrival to the ED, the patient had a pulse of 74 bpm, respiratory rate 23 breaths per minute, a BP of 122/65 mmHg, SaO_2_ 83%. During the primary survey, the King LT was removed and the patient was re-intubated with an endotracheal tube on the first attempt using direct laryngoscopy. He was pre-oxygenated with saturations maximizing in the mid-80s. Secondary survey findings were significant for a gunshot wound to the right temporal region. No additional injuries were found. Pupils were three millimeters bilaterally and fixed, weak corneal reflex, absent cough and gag reflex, and decerebrate posturing in all extremities. Head computed tomography revealed a right parietal entry wound with fragments scattered through the bullet tract and to the left of midline, a large subdural hematoma with rightward shift, diffuse cerebral edema, and a comminuted skull fracture. A chest radiograph (CXR) revealed widening of the superior right mediastinum with loss of definition of airspace in the right upper lobe and absence of the minor fissure, most consistent with complete collapse of right upper lobe (Image). Upon reexamination, no additional injuries or entry sites were found to correlate with the radiograph findings.

The patient was given 100 grams of mannitol for bradycardia and signs of herniation and one grams of levetiracetam prior to transfer to the intensive care unit (ICU) for expectant management of his head injury while waiting for family to arrive. At admission to ICU, his blood pressure was 80/50 mmHg with heart rate in the seventies. His neurologic exam remained poor. Bronchoscopy was performed in the ICU due to persistent hypoxia and revealed blood obstructing the right mainstem bronchus, which was suctioned and evacuated from the right lung. A right-sided chest tube was placed for pneumothorax identified after bronchoscopy with blood evacuated from the chest cavity. Due to the poor prognosis of the patient, care was transitioned to comfort measures and he was compassionately extubated.

Autopsy was performed approximately 12 hours after death. In addition to significant intracranial hemorrhage and edema, the patient was noted to have a transection of the gastroesophageal junction and a large disruption of the greater curvature of the stomach. Blood was noted in the mediastinum and within the pleural and peritoneal cavities. No inflammation or ischemic changes were noted on histologic specimens of the stomach, but specimens from the esophagus and gastroesophageal junction were suggestive of ischemia.

## DISCUSSION

Rupture of the esophagus and stomach are rare complications of esophageal or failed intubation. These injuries are likely under-recognized and under-reported. Esophageal or gastric rupture is more often associated with cardiopulmonary resuscitation (CPR), occurring in up to 12% of those who undergo CPR.^3^ In the case described, no CPR was performed, but he did have a failed intubation attempt.^4^ The use of bag-valve-mask ventilation, especially if improperly performed or performed after esophageal intubation, can greatly increase pressure in the stomach, with as little as 15 cm H_2_O resulting in gastric distention. This distention decreases the amount of air that is able to leave the stomach, resulting in an increase in pressure and subsequent rupture.^3^ Risk factors for perforation are similar to risk factors for difficult intubations, including poor visualization, macroglossia, trismus, and short neck.^2^ Other potential causes of esophageal rupture could be direct trauma from the endotracheal tube or the esophageal airway placement, but the location of the perforation make direct trauma unlikely in this case.

**CPC-EM Capsule**

What do we already know about this clinical entity?

Esophageal perforation is rare but with significant mortality if not quickly identified. Iatrogenic and spontaneous ruptures are the most common causes.

What makes this presentation of disease reportable?

Esophageal rupture secondary to esophageal intubation is a rare event, but the conditions predisposing patients are common in the emergency department (ED) and it should remain a consideration for ED practitioners. We found no case reports of rupture secondary to prehospital intubation.

What is the major learning point?

Esophageal intubation can be recognized through a combination of radiography findings, clinical findings, and historical features.

How might this improve emergency medicine practice?

Recognition of esophageal perforation after intubation will improve emergency physicians’ ability to initiate early evaluation, consultation, and therapy in affected patients.

Typical findings suggestive of esophageal rupture on chest radiography are a widened mediastinum and pneumomediastinum. Subcutaneous emphysema and loss of contour of the descending aorta can also be visualized.^2^ CXR using water-soluble contrast can reveal sites of contrast leak and can be repeated if initial imaging is negative but clinical suspicion remains elevated.^5^ Esophageal tears are typically visualized in the upper one-third of the esophagus or the distal esophagus near the esophagogastric junction.^2^ Subtle findings such as neck pain or dysphagia may be noted. Objective signs such as neck swelling or subcutaneous emphysema may be recognized. More significantly, signs of sepsis can appear quickly if the injury is missed.^2^,^6^

In the controlled surgical environment, patients may be able to report symptoms postoperatively; however, our patient remained intubated and sedated and the injury was not discovered until postmortem. On autopsy, the significant amount of hemorrhage present supported the assertion that the injury occurred before death. A failed intubation attempt in the field, abnormal mediastinum visualized on chest radiography, and treatment-resistant hypoxia suggest that this injury was likely present at the time of ED evaluation but was not recognized.

Treatment is controversial due to low incidence and very high mortality with little outcomes data guiding management. Early recognition and aggressive antimicrobial therapy is likely associated with best outcomes, with mortality rising sharply after the first 24 hours (over 60%), typically due to overwhelming infection.^1^ Patients who are clinically stable may be managed in a non-operative fashion, but unstable patients require primary surgical repair or esophageal stent placement. Early recognition is key to improving clinical outcomes.^5^

An alternative diagnosis that can cause gastroesophageal perforation is postmortem gastromalacia and esophagomalacia, which are well documented in the literature. Endogenous enzymes, pepsinogen and hydrochloric acid are released leading to autolytic rupture with subsequent effects including pneumoperitoneum or pneumothorax.^7^ Although it can be difficult to discern timing of the rupture, gastromalacia is more common in the fundus or distal esophagus, with 70–80% of perforations occurring at the lesser curvature of the stomach.^4^ It is typically recognized as thinned tissue without inflammation, hemorrhage, or signs of peritonitis.^7^ The timing of these changes after death remains unclear, but it is reported to occur within the first 20 hours following death.^7^,^8^ In this case the patient’s clinical presentation, recognized antemortem pneumothorax, and postmortem evidence of hemorrhage suggest an antemortem injury, most likely secondary to intubation trauma and esophageal insufflation.

## CONCLUSION

In conclusion, this case displays a rare complication of a common procedure in the setting of trauma in the prehospital environment. Rapid recognition of esophageal or gastric perforation can be critical to initiating early therapy and limiting the morbidity and mortality of this serious condition.

Documented patient informed consent and/or Institutional Review Board approval has been obtained and filed for publication of this case report.

## Figures and Tables

**Image:**
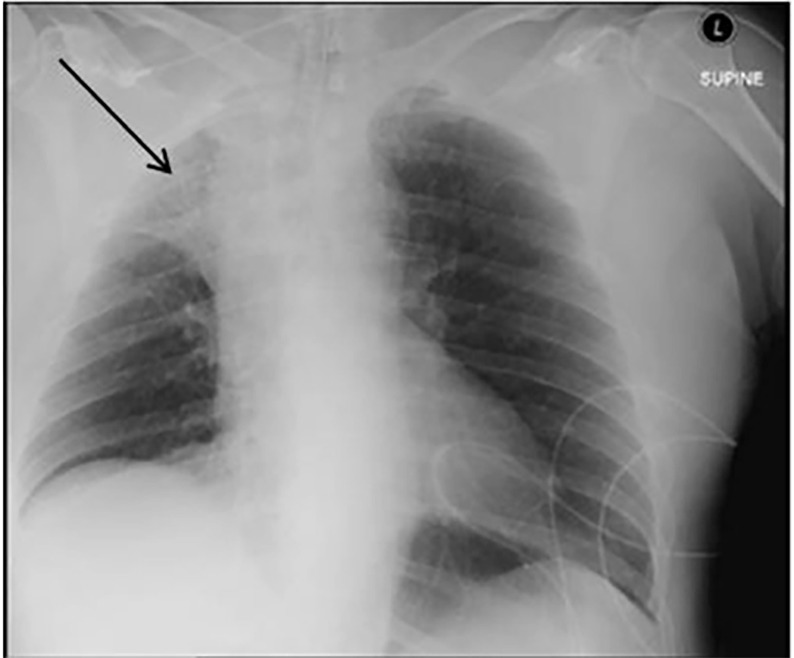
Initial chest radiograph demonstrating mediastinal widening and collapse of the right upper lobe (arrow) in patient with esophageal perforation
